# AI-Based Homology Modelling of Fatty Acid Transport Protein 1 Using AlphaFold: Structural Elucidation and Molecular Dynamics Exploration

**DOI:** 10.3390/biom13111670

**Published:** 2023-11-20

**Authors:** Ranjitha Acharya, Shilpa S. Shetty, Gollapalli Pavan, Flama Monteiro, Manne Munikumar, Sriram Naresh, Nalilu Suchetha Kumari

**Affiliations:** 1 Department of Biochemistry, KS Hegde Medical Academy, Nitte (Deemed to be University), Mangalore 575018, India; ranjitha.acharya9988@gmail.com (R.A.); mascarenhasflama@gmail.com (F.M.); sriramnaresh123@gmail.com (S.N.); 2 Central Research Laboratory, KS Hegde Medical Academy, Nitte (Deemed to be University), Mangalore 575018, India; shilpajshetty@nitte.edu.in (S.S.S.); gollapallipavan@nitte.edu.in (G.P.); 3 Clinical Division, ICMR-National Institute of Nutrition, Jamai-Osmania (Post), Hyderabad 500007, India; mannemunikumar.bioinfo@gmail.com

**Keywords:** molecular dynamic simulations, FATP1, LCFA, VLCFA

## Abstract

Fatty acid transport protein 1 (FATP1) is an integral transmembrane protein that is involved in facilitating the translocation of long-chain fatty acids (LCFA) across the plasma membrane, thereby orchestrating the importation of LCFA into the cell. FATP1 also functions as an acyl-CoA ligase, catalyzing the ATP-dependent formation of fatty acyl-CoA using LCFA and VLCFA (very-long-chain fatty acids) as substrates. It is expressed in various types of tissues and is involved in the regulation of crucial signalling pathways, thus playing a vital role in numerous physiological and pathological conditions. Structural insight about FATP1 is, thus, extremely important for understanding the mechanism of action of this protein and developing efficient treatments against its anomalous expression and dysregulation, which are often associated with pathological conditions such as breast cancer. As of now, there has been no prior prediction or evaluation of the 3D configuration of the human FATP1 protein, hindering a comprehensive understanding of the distinct functional roles of its individual domains. In our pursuit to unravel the structure of the most commonly expressed isoforms of FATP1, we employed the cutting-edge ALPHAFOLD 2 model for an initial prediction of the entire protein’s structure. This prediction was complemented by molecular dynamics simulations, focusing on the most promising model. We predicted the structure of FATP1 in silico and thoroughly refined and validated it using coarse and molecular dynamics in the absence of the complete crystal structure. Their relative dynamics revealed the different properties of the characteristic FATP1.

## 1. Introduction

The solute carrier family 27 (*SLC27A*), also known as fatty acid transport proteins (FATPs), is a family of transmembrane proteins made up of six isoforms (FATP1-6). Its primary function revolves around the facilitation of the cellular uptake and translocation of long-chain fatty acids (LCFAs) across the plasma membrane. They differ in molecular mass, degree of posttranslational modification, and intracellular localization. Some show a distinctive pattern of tissue distribution, while others are ubiquitously expressed [1]. FATPs possess essential functional domains, including an intracellular acyl-CoA synthase activity site, an extracellular fatty acid-binding site, and an ATP-binding domain [2]. These distinctive structural features endow FATPs with the capability to effectively transport LCFAs into cells.

The *SLC27A1* gene exhibits a remarkable degree of evolutionary conservation across diverse biological species, spanning from bacteria to mammals, and is classified among the candidate fatty acid transporters [3,4,5,6].

FATPs are found in various organisms, including *Mycobacterium tuberculosis*, *Saccharomyces cerevisiae*, *Fugu rubripes*, *Caenorhabditis elegans*, and *Drosophila melanogaster.* The *C. elegans* and Mycobacterium FATPs, when overexpressed in COS (fibroblast-like cell line African green monkey kidney) cells increase LCFA uptake, demonstrating that the function of the FATP gene family has remained constant throughout evolution [7]. Fatty acids gain entry into cells through the FATP transporters, subsequently undergoing a transformation into metabolites including ceramide, diacylglycerol, and inositol phospholipid derivatives. These metabolites assume the role of second messengers within numerous cellular regulatory systems [8]. They serve pivotal functions within signalling pathways that govern cellular processes, encompassing apoptosis, inflammation, and insulin sensitivity. Among the six isoforms of FATPs, FATP1 stands out, acknowledged for its involvement in lipid accumulation within the adipose tissue [9,10]. Moreover, it has been demonstrated that the expression of FATP1 spans across diverse tissues, and it is involved in the metabolic processing of fatty acids in the heart, skeletal muscle, kidney, lung, skin, adipose tissue, and brain [11,12,13,14,15,16,17,18].

There is an indication that this specific protein might contribute to obesity-related diseases such as breast cancer, and subsequently, insulin resistance and the onset of type 2 diabetes [19]. Furthermore, studies have shown that elevated levels of FATP1 in breast cancer cells are associated with increased tumour growth and metastasis [20,21,22]. This suggests that targeting FATP1 could potentially be a promising therapeutic strategy for both breast cancer and the prevention of insulin resistance and type 2 diabetes. In the altered yeast homolog of FATP1, very-long-chain acyl CoA synthase expression was reduced [23]. The overexpression of wild-type FATP1 in fibroblast-like (COS1) cells boosted acyl-CoA synthetase (ACS) activity, whereas ACS activity was eliminated in the FATP1 mutant region, which is highly conserved in ACS proteins [24]. According to studies, overexpressed FATP1 in 3T3-L1 fibroblasts caused an increase in the LCFA uptake [25]. When inhibited, overexpressed FATP1 reduced the lipid uptake in melanoma cells, which displayed unique lipid accumulation [26]. A fascinating study discovered that fatty acid transport protein 1 plays an important role in the transfer of fatty acids in the tumour microenvironment between breast cancer cells (BCCs) and noncancerous cells. FATP1 inhibition appears to be a viable treatment method since it raised FATP1/*SLC27A1* expression in human triple-negative breast cancer, which was associated with a significant reduction in overall survival [27].

In the context of humans, mutations within the FATP1/SLC27A1 gene have been firmly linked to metabolic disorders, such as insulin resistance and type 2 diabetes, stemming from compromised cellular fatty acid uptake and utilization. Additionally, investigations have unveiled that FATP1 expression is markedly upregulated in breast cancer cells, hinting at a potential role in tumour growth and progression [20,28]. FATP1 is a 646 amino-acid integral transmembrane protein with a single membrane-spanning domain, a short extracellular/luminal section, and a longer intracellular segment. Both the AMP-binding region and the ACSL catalytic domain are situated within the intracellular region, as reported in reference [29]. FATP1 plays a pivotal role in facilitating the translocation of long-chain fatty acids (LCFA) across the plasma membrane, thereby orchestrating the importation of LCFA into the cell [30]. This physiological process assumes critical importance in the cellular energy metabolism, given that LCFA represents a vital source of fuel.

Notably, within the context of protein expression, it is noteworthy that FATP1 exhibits predominant expression patterns in breast cancer and skin cancer [26,27]. FATP1 can act directly as a true transporter, or alternatively, in a cytoplasmic or membrane-associated multimeric protein complex to trap and draw FAs towards accumulation. FATP1 also functions as an acyl-CoA ligase, catalyzing the ATP-dependent formation of fatty acyl-CoA using LCFA and VLCFA (very-long-chain fatty acids) as substrates [31,32,33,34]. The expression of FATP1 in cancer cells suggests its potential role in tumorigenesis and cancer progression [35]. In addition, studies have shown that the inhibition of FATP1 can reduce cancer cell proliferation and migration, making it a promising target for cancer therapy [20,36]. In tissues undergoing high levels of beta-oxidation or triglyceride production, FATP1 plays a crucial function in controlling the exogenous LCFA substrates that are readily available. It is also likely known to play a role in the transfer of FAs through the blood–brain barrier [37].

FATPs have been shown to play important roles in lipid metabolism and energy homeostasis. They are also implicated in various lipid-related disorders and metabolic diseases such as obesity, diabetes, and cancer. Therefore, understanding the function and regulation of FATPs is crucial for developing treatments for these diseases. This knowledge could ultimately lead to the development of new therapies and preventative measures. Furthermore, understanding how FATPs work could help improve our understanding of cell metabolism and energy balance.

Although FATP1′s multifunctionality has been widely acknowledged, the 3D structure of the human FATP1 protein has not yet been predicted or assessed to fully comprehend the functional implications of the FATP1 domains individually. Therefore, an attempt was made to understand the comprehensive role of FATP1 in the development of metabolic diseases including cancer through an in silico structural analysis of FATP1 and a protein–protein interaction (PPI) network analysis.

## 2. Methods

### 2.1. In Silico Protein Modelling

The crystal structure of the human FATP1 protein (Q6PCB7) was not available in the Protein Data Bank (PDB) (https://www.rcsb.org/, accessed on 20 April 2022). Consequently, we employed a tool known as ALPHAFOLD, a comprehensive database, for the purpose of protein structure modelling. ALPHAFOLD, an artificial intelligence (AI) system developed by DeepMind, leverages amino acid sequences to generate state-of-the-art forecasts pertaining to protein structures [38]. The CASP (Critical Assessment of Structure Predictions) stands as a biennial challenge designed for researchers to rigorously assess the accuracy of their predictive models against empirical experimental-derived structures. In the context of CASP14, the organisers bestowed recognition upon AlphaFold as an innovative solution to the intricate problem of predicting protein structures back in the year 2020 [39]. AlphaFold’s unparalleled precision and remarkable computational speed have enabled the rapid and efficient assembly of an extensive database housing structure predictions. This development holds the promise of revolutionising the field of biology by granting researchers the ability to access structural models for nearly any given protein sequence. Consequently, this transformative capability is poised to reshape the way biologists approach their research endeavours and expedite the progression of their projects. The AlphaFold methodology, along with the invaluable insights garnered from predictions encompassing the entirety of the human proteome, have been comprehensively elucidated in recent publications [40,41,42].

### 2.2. Secondary Structure Prediction

Secondary structural patterns were examined using the SOPMA (https://npsa-prabi.ibcp.fr/cgi-bin/npsa automat. pl?page=npsasopma.html, accessed on 11 August 2022) and PSIPRED (http://bioinf.cs.ucl.ac.uk/psipred/, accessed on 16 August 2022) servers [43,44]. The GalaxyRefine server (http://galaxy.seoklab.org/index.html, accessed on 16 August 2022) was used to optimise and refine. The 3D-modelled FATP1 improved the clash scores, poor rotamers, Ramachandran outliers (%), and bad side chain rotamers [45]. The final model was validated further using the HARMONY (http://caps.ncbs.res.in/harmony/, accessed on 16 August 2022) and ProSA web servers (https://prosa.services.came.sbg.ac.at/prosa.php, accessed on 16 August 2022). Based on previously characterised structures, HARMONY was used to determine global and local errors in the protein’s 3D conformation. The HARMONY substitution graph provided smoothed scores between query sequences compared to reverse sequences (as controls) to detect possible local errors in regions where the reverse sequence has a higher substitution score [46]. A web-based tool that highlights protein structure errors based on energy plots and Z-scores derived from conformational variations regarding experimentally derived structural patterns [47]. Backbone flexibility at the residue level was predicted by the DynaMine (http://dynamine.ibsquare.be/, accessed on 16 August 2022) server in the form of backbone N-H S2 order parameter values. These S2 values represented the atomic bond vector’s movement restrictions in the molecular reference frame. DynaMine’s N-H S2 order parameter values were derived directly from experimentally determined NMR chemical shifts [47].

### 2.3. Coarse Dynamics’ Refinement and Residue Level Propensity

To determine the residue level fluctuations concerning the most favourable conformation of the protein, a coarse dynamics study was performed using the CABS flex server (http://biocomp.chem.uw.edu.pI/CABSflex2/, accessed on 17 August 2022) as a simulation engine. CABS flex employs a coarse-grained protein model. The stability was determined from the root mean square fluctuation (RMSF) trajectories at the nanomolar (nm) scale, which were produced based on default constraints that gave pair atoms maximum and minimum ranges for undergoing their specific dynamic orientations within defined spaces. The fluctuations outside of the assigned ranges were penalised and labelled as unstable. In an aqueous environment generated by an all-atom molecular dynamics’ simulation (10 ns timescale with appropriate force fields), the default settings and constraints were optimised to combine coarse dynamics’ simulations and consensus protein fluctuations. The default mode was selected with minimum and maximum conformational distances of 3.8 and 8.0 Å, respectively; gap = 3 (minimum distance between the previous and next amino acid in the chain to be restrained).

### 2.4. Model Validation

PROCHECK [48] ERRAT [49], VERIFY 3D [50], and PROVE [51] tools from the structural analysis and verification server (SAVES) (http://nihserver.mbi.ucla.edu/SAVES, accessed on 19 May 2022) were used to validate the 3D model of FATP1. The stereochemical quality of the protein structure was evaluated using PROCHECK. The Verify3D tool evaluated the 3D protein structure concurrently by determining whether an atomic model (3D) and its amino acid sequence (1D) were compatible. The ProSA web servers (https://prosa.services.came.sbg.ac.at/prosa.php, accessed on 19 May 2022) were used to host the predicted models [45]. Based on the C-alpha atoms in the input structure, ProSA created an overall quality score and validated a low-resolution structure for approximation models for the specific PDB structure of the FATP1 protein. The Z-score that was generated from the plot during structure prediction serves as a measure of the model’s quality.

### 2.5. Protein Stability Analysis through Molecular Dynamic Simulation (MDS)

The MDS analysis governs the essential knowledge of a protein’s stability in a simulated cellular environment (aqueous or lipid) over a favourable period. The MDS for the FATP1 refined structure was run in the current study for 100 ns. The GROMACS 2018 (1.6) package with the GROMOS96 43 A1 force field was used to simulate the protein in an aqueous environment. The protein was placed in the centre of a cubic box with uniform edge distances (1.5 nm), and it was solvated using a simple point-charge water model. It was neutralised by adding the necessary counter ions (Na^+^ or Cl^−^) [52]. Energy minimisation with 50,000 steps and a 1000 kJ/mol/nm convergence tolerance was performed using the steepest descent algorithm. Standard NVT (number of particles, volume, and temperature) and NPT (number of particles, pressure, and temperature) ensembles were used to equilibrate the system for 150 ps. To equilibrate the system for 150 ps, standard NVT and NPT groups were utilised. A particle-mesh Ewald electrostatics summation was used to handle long-range electrostatic interactions with an order of 4.0 and a Fourier spacing of 0.16 nm. By using motion equations on the box vectors, the Parrinello–Rahman extended coupling ensemble was used for pressure scaling [53,54]. The RMSD analysis, which measures the variations in the protein trajectory concerning its backbone, was used to infer the stability of FATP1. Higher average RMSF values revealed the average positional dynamicity of each atom along the entire MDS, whereas lower average RMSF values indicated excellent stability. The Radius of Gyration (Rg) study [55,56] calculated the weighted RMS distances of the protein backbone atoms depicted by Rg (nm) versus the overall run duration to determine the integrity of the simulated FATP1 structure. Using INTERPRO, the functional domains of the proteins were screened to examine the relationship between structure and function.

### 2.6. Protein–Protein Interaction (PPI) Network Analysis

Understanding the dynamic process by which genes and proteins function is of paramount importance. In this context, the STRING database (accessible at https://string-db.org/, accessed on 19 May 2022) was utilised to build a protein–protein interaction network [57,58]. The protein names of the prioritised genes were employed as seeds for constructing the protein interaction (PPI) network, encompassing all genes, proteins, and their neighbouring interactions [59]. These seed proteins played a pivotal role in generating network connections involving human proteins. The construction of this network took into consideration various sources of information, including experimental validation, text mining, databases, co-expression patterns, and neighbourhood interactions, all at a high confidence threshold of 0.7.

### 2.7. Gene Set Enrichment and Pathway Analysis

The critical biological processes of the achieved targets were examined using functional Gene Ontology (GO) enrichment [60]. The functionally organised pathway term network was built by combining the GO terms and Kyoto Encyclopaedia of Genes and Genomes (KEGG) pathways. The parameters set forth for gene list enrichment included the statistical test enrichment/depletion (two-sided hypergeometric test), the correlation test-Bonferroni step-down), the minimum GO level 3, the maximum GO level 8, the Kappa score threshold of 0.4, the GO fusion false, the GO group-true, and *p* ≤ 0.05. ShinyGO is a user-friendly, graphical tool for enrichment analysis that helps researchers decipher the biological significance of a long list of genes [61]. This study used the KEGG database with a significance level of (*p* = 0.05) [62].

## 3. Results

### 3.1. MDS-Based In Silico Structure Prediction and Validation

**Figure 1:**
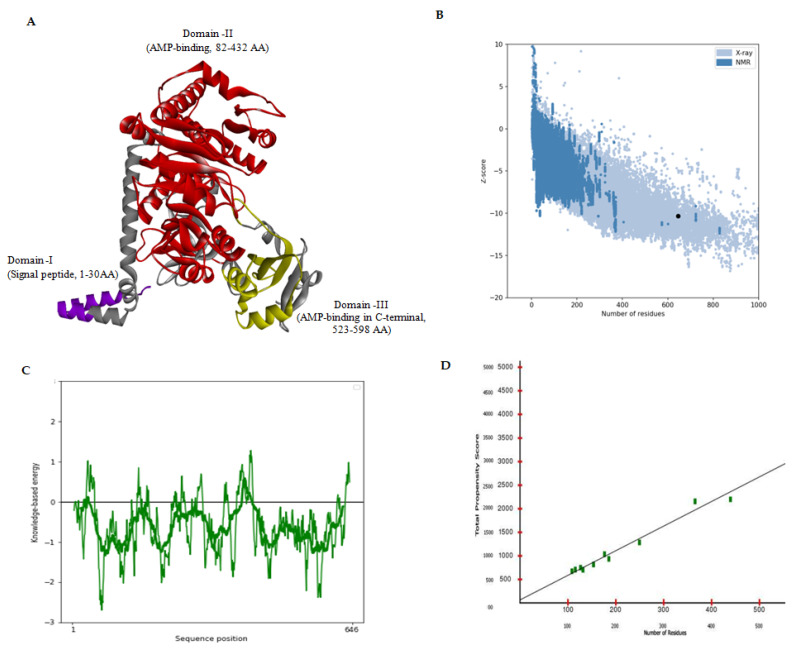
Structural features of FATP1. (**A**) Refined modelled structure of FATP1 with domains. (**B**) Global model quality of FATP1. (**C**) Local model quality of FATP1. (**D**) Propensity plot of FATP1.

It is well-known that different amino acids have different environmental preferences and are crucial for preserving proteins’ structural stability [63,64,65,66,67]. Based on the secondary structure prediction, it was discovered that the human FATP1 protein had solvent accessibility at 330 area/(nm^2^). The secondary structure profile for the FATP1 domain, which was primarily composed of random coils (33.44%), followed by helix (34.98%), extended strand (22.91%), and turns (8.67%), was consistent with the fact that 16% of the residues were buried (Supplementary Figure S1). The 3D model of the FATP1 protein was created and put through several validations and optimisations (Figure 1A). In the secondary structure prediction, we have obtained three domains, and the signal peptide region (Domain I), which ranges in length from 1 to 30 amino acids, is one of the three domains we acquired from the secondary structure prediction. The AMP-dependent synthase/ligase domain was situated in the region between amino acids 82 and 432 (Domain II), and amino acids 523 to 598 (Domain III) was where the AMP-binding enzyme was found in the C-terminal region (Figure 1A).

**Figure 2:**
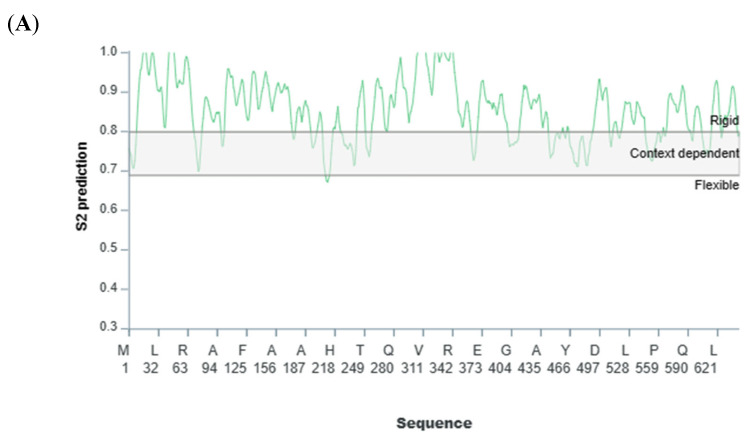

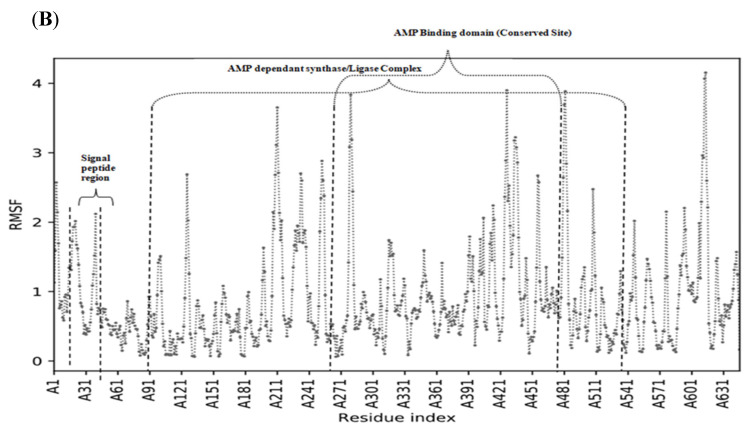
Stability analysis of FATP1. (**A**) Backbone stability profile of FATP1. (**B**) Residue-level fluctuation profile of FATP1 structure (fatty acid transport protein 1).

In the refined model, it was observed that 94.4% of the residues residing within the Ramachandran-favoured region exhibited a prevalence of weak rotameric conformations, suggesting a high degree of conformational stability in the protein structure. The final predicted structure of the FATP1 was submitted to the Protein Model Database (PMDB) and given the PMDB Id: PM0084554). FATP1 had a Z-score of −10.33, which was the highest among the experimentally determined (X-ray diffraction) proteins (Figure 1B). The ProSA web energetic curve for the entire FATP1 protein and its functional domains was significantly below the stability threshold value (0.00), indicating that they were in a stable conformation (Figure 1C). FATP1 was close to the straight fit line of the propensity calibration plot, and high propensity scores indicated that there were few structural-related error areas (Figure 1D). The query and reverse sequences had nominal intersections on the FATP1 substitution curve, indicating small local errors (Supplementary Figure S2). Additionally, the average backbone rigidity, predicted by the human FATP1 model, was remarkably stable (S2 > 0.8) (see Figure 2A). According to coarse dynamics’ simulation, the final refined structure’s average RMSF was 0.64, indicating that average residue fluctuation was minimal (Figure 2B).

### 3.2. Structural Stability of Fatty Acid Transport Protein 1

**Figure 3:**
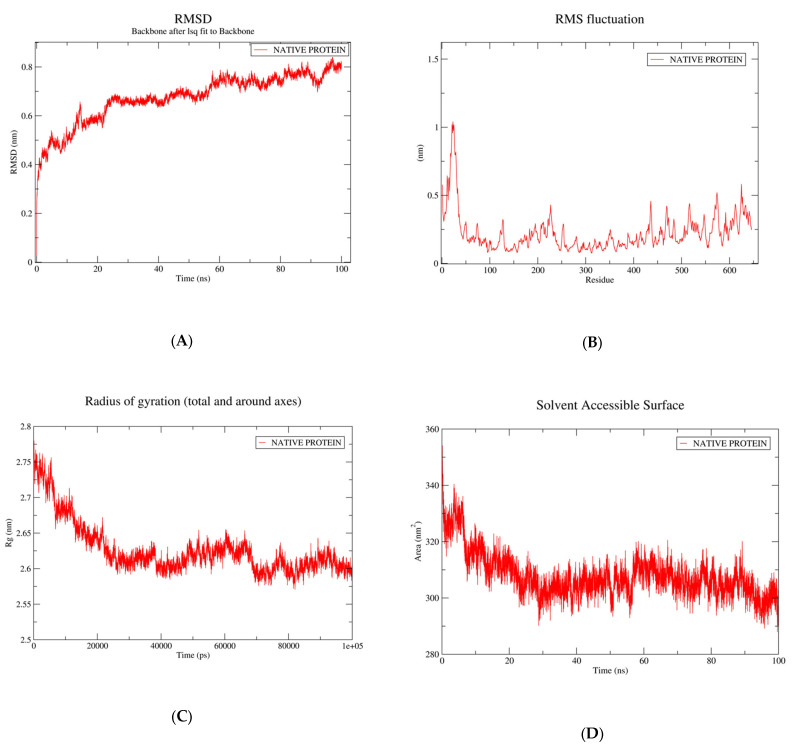
MDS analysis of FATP1: (**A**) RMSD trajectory of FATP1; (**B**) atomic-level RMSF of FATP1; (**C**) Rg curve of FATP1. (**D**) The solvent accessible surface of FATP1, fatty acid transport protein 1; Rg, radius of gyration.

When exposed to MDS for a duration of 100 ns within an aqueous milieu, the verified structural configuration of FATP1 exhibited an intriguing behaviour. The RMSD trajectory depicted a distinctive characteristic, as it attained equilibrium shortly after the 20 ns mark, demonstrating a consistent, minimal fluctuation range between 2.4 and 2.6 nm. Notably, this stability persisted throughout the entirety of the MDS (Figure 3A). It was also mentioned that the typical RMSF value, which denoted minimal atomic level positional variation, was between 0.05 and 1.0 nm (Figure 3B). The protein structure’s compactness was ensured by Rg’s low average value (2.6 nm) and steady trajectory (Figure 3C). With a mean stability of 5.43 × 10^6^ kJ/mol, the FATP1 potential energy curve (Figure 3D) also demonstrated a constant energy profile during MDS. The MDS analysis provided additional confirmation of the stability traits of the FATP1 model that we had anticipated. It was discovered that the free proteins’ RMSD immediately increased and reached 0.65 nm (6.5 Å) at 15 ns, while the RMSD gradually increased to 0.4 nm and then slowly started decreasing. After that, the RMSD gradually rose for the later duration of the simulation before slowly falling again. It then reached a plateau and remained stable for 100 ns. At the plateau time, the later differential dynamic behaviour gave FATP1 a more secure and constrained accommodation, and nearly the entire protein was found to be stable for 100 ns.

### 3.3. Root-Mean-Square Fluctuation and Protein Flexibility of Fatty Acid Transport Protein 1

The backbone RMSF of FATP1 was determined separately using the GROMACS “gmx_rmsf” command line. This flexibility validation criterion provides details on the contribution of particular protein residues to the structural fluctuations of the protein. Each residue’s average departure from its reference point inside the reduced starting structures is determined by the RMSF over time [68]. A “ΔRMSF cut-off value of 0.30 was used to evaluate the significance of changes in structural movements, and residues with values higher than 0.30 were assumed to be less mobile” [69]. Figure 3B’s outcomes demonstrated typical terminal-free residue behaviour with significantly low RMSF values. This is because terminal-free residues are more likely than core residues to fluctuate at the highest deviations, which is what is typically seen in well-behaved MD simulations. The RMS fluctuation profile started at 0.5 nm, decreased gradually at the first residue, increased steadily to 1 nm up until the 40th residue, and then dramatically decreased again until the 50th residue. Furthermore, no additional fluctuations were noticed. However, the protein region with the greatest fluctuation was found to be the C-terminal region. This region of the fatty acid transport protein 1 [Uniprot Id: Q6PCB7 S27A1_HUMAN] had topological domains (1–13 residues) in the extracellular region and transmembrane domains (14–34 residues) in the helical region, suggesting greater flexibility during MD simulation. This observation may have implications for the function of the protein, as increased flexibility in this region could potentially aid in the transport of fatty acids across cell membranes. Further studies could investigate the relationship between protein flexibility and its role in lipid transport.

### 3.4. Radius of Gyration

To determine the complex liability, the Rg profile of the simulation systems was also calculated. The lower mobility of the complex was indicated by the Rg trend of the amylase systems, which exhibited steady-state behaviour and little change. Utilising the GROMACS “gmx gyrate” command script, the Rg was tracked throughout the entire MD trajectory to gain a deeper understanding of the complex stability under investigation. This stability metric considers the stability of the ternary structure of the protein, where Rg is the mass-weighted root-mean-square deviation (RMSD) for a collection of atoms with a common mass centre [70]. So, it would be possible to determine the extended stability/compactness of the investigated molecule by observing low Rg values that eventually plateau at an average value. The investigation’s measured Rg proved that the FATP1 preferred stability (Figure 3C). The protein structure’s compactness was made possible by Rg’s low average value (2.6 nm) and steady trajectory. It suggested that during the MD simulation, the protein was adopting a more compact structure and achieving greater stability. This finding is consistent with previous studies that have shown a correlation between low Rg values and increased stability in proteins. It also highlights the importance of Rg as a valuable tool for investigating a protein’s structure and stability. Furthermore, the observed stability and compactness of the protein structure may have important implications for its function and interactions with other molecules in the cell. Future studies could investigate how changes in environmental conditions or mutations in the protein sequence affect its stability and compactness.

### 3.5. Calculation of Solvent Assessable Surface Area (SASA)

To understand how the protein surface area varies, the SASA complexes were studied. The SASA was correlated with protein volume and surface area The simulation demonstrated that the FATP1 had a higher SASA and a stronger affinity for the ligand molecules. The FATP1 complexes reached a stable state after 40 ns and remained rigid for the remaining 100 ns of the simulation session (Figure 3D). These findings suggest that the SASA measurement can be used as a reliable indicator of protein–ligand interactions and stability. Further studies could explore the potential of using the SASA as a screening tool for drug discovery and development. 

### 3.6. Construction of Protein–Protein Interaction (PPI) Network

**Figure 4:**
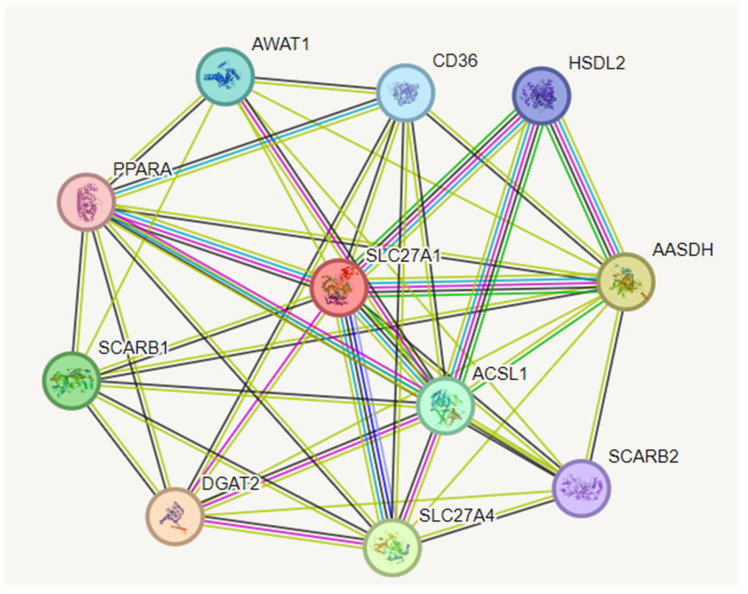
Construction of protein–protein interaction (PPI) network.

We have used the STRING database v9 (Search Tool for the Retrieval of Interacting Genes, available at: http://string-db.org/, accessed on 19 May 2022 [65]) with the protein names of the prioritised genes as seeds for the construction of the protein interaction (PPI) network. This included all genes/proteins and all neighbour interactions [66]. To begin, network interactions connected with human proteins were created based on the seed proteins. With a high confidence level of 0.7, the interactions were calculated using text mining, experiments, databases, co-expression, neighbourhood, gene fusion, and co-occurrence [67]. The number of edges connected to the assigned node was a high degree, indicating the significance of the protein in biological interactions (Figure 4). The average node degree of the network showed 7.82 with an expected number of edges of 43. A characteristic feature of a protein–protein interaction network is that it is characterised by a small number of highly connected nodes and the remaining node with few interactions. Genes from a list can be displayed on pathway maps to aid the biological interpretation in a network setting.

### 3.7. Interactomics Analysis of Hub Gene

Hub genes, also known as required modules with a higher degree of interconnectivity, are critical for understanding the ways of biological networks. The functional importance of the cellular map in drug target selection was investigated using an interactomics analysis, which displayed molecular interaction networks with physical connections between neighbours [71]. We were able to estimate the top hub gene near the neighbourhood ranking network for addressing the novel function of the gene in the context of biological reactions using the Biological General Repository for Interaction Datasets (BioGRID 4.4; Canada). Physical relationships and degree evidence were considered for identifying hub gene networks [72].

**Figure 5:**
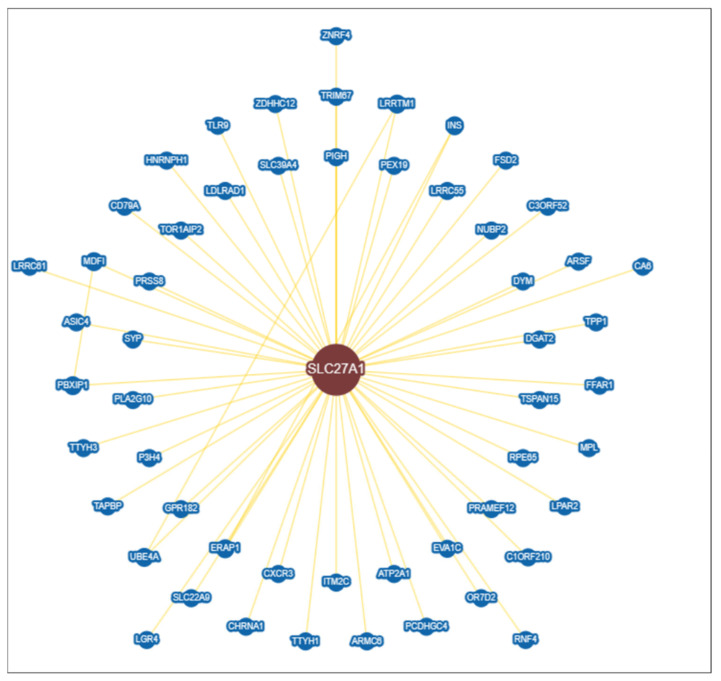
Interactomic analysis of hub gene showing the network of FATP1 with various proteins.

The top gene ontology biological process shown by the BioGRID version 4.4.226 includes (https://thebiogrid.org/132009/summary/homo-sapiens/slc27a1.html, accessed on 30 september 2023) the cardiolipin biosynthetic process, cellular lipid metabolic process, long-chain fatty acid transport, negative regulation of phospholipid biosynthetic process, phosphatidic acid biosynthetic process, phosphatidylcholine biosynthetic process, phosphatidylethanolamine biosynthetic process, phosphatidylglycerol biosynthetic process, phosphatidylinositol biosynthetic process, phosphatidylserine biosynthetic process, small molecule metabolic process, and transmembrane transport. The gene ontology cellular components involved the membrane and plasma membrane, which showed about 54 interactors (Supplementary Table S1) and 55 interactions which are mentioned in Supplementary Table S2. Out of these, 52 high throughput and 3 low throughput interactions were observed. The Biological General Repository for Interaction Dataset showed a network of Slc27a1 with various proteins, which can be visualised (Figure 5).

### 3.8. GO Enrichment Analysis

A series of events that are put together by one or more molecular functions are described by the GO biological process (BP), molecular function (MF), and cellular component (CC) terms. In total, 53 GO-BP, 12 GO-MF, and 33 GOCC terms were enriched. Table 1 and Table 2 display the most important GO-BP, GO-MF, and GO-CC terms. Some of the prominent biological processes (Table 1) that might show the involvement of FATP1 in various metabolic diseases including breast cancer progression were the regulation of lipid metabolic process [73,74], cellular response to fatty acid [75], and positive regulation of the triglyceride biosynthetic process. Previous studies have reported the involvement of FATP1 in cell proliferation [76], lipid localisation, the hormone-mediated signalling pathway, lipid transport, response to oleic acid, long-chain fatty acid transport into cell [10], cellular response to hormone stimulus, intracellular receptor signalling pathway, positive regulation of the triglyceride biosynthetic process, positive regulation of transcription, regulation of fatty acid oxidation, glycerolipid biosynthetic process, and response to progesterone. These findings suggest that the FATP1 is intricately linked to various lipid-related processes, hormone-mediated signalling pathways, and cellular responses to specific stimuli. The involvement of FATP1 in transporting LCFAs into cells, intracellular receptor signalling pathways, and regulation of fatty acid oxidation highlights the importance of FATP1 in metabolic diseases including cancer. Since breast cancer is a hormone-sensitive cancer, the response to progesterone and other hormones emphasises the role of FATP1 in controlling breast cancer. Additionally, studies have shown that high levels of FATP1 expression are associated with increased tumour growth and aggressiveness, highlighting its significance in the progression of hormone-sensitive breast cancer [10]. Overall, these processes suggest that FATP1 may play a role in regulating lipid metabolism, responding to fatty acids, and promoting cell proliferation in breast cancer progression.

Important molecular functions that are listed in Supplementary Table S3 include nuclear receptor transcription co-activator activity, transcription regulator activity, nuclear receptor binding, RNA polymerase II-specific DNA-binding transcription factor binding, arachidonate-CoA ligase activity, RNA polymerase II intronic transcription regulatory region sequence-specific DNA binding, and chromatin binding. The binding activities mentioned, such as DNA binding and chromatin binding, suggest that FATP1 is involved in regulating gene expression. This is supported by its RNA polymerase II-specific DNA-binding transcription factor binding activity, indicating its role in controlling the transcription of specific genes. Additionally, the arachidonate-CoA ligase and long-chain fatty acid-CoA ligase activities suggest that FATP1 is responsible for activating these fatty acids for further metabolic processes. Overall, the diverse molecular functions and cellular components associated with FATP1 demonstrate its importance in various biological processes and shed light on its functional properties.

Out of 33 various cellular components mapped against the enriched set (GO:0071564), perinuclear prominent terms were revealed by the PANTHER overrepresentation test (released 20221013). These terms represent various cellular compartments and structures. They include the perinuclear endoplasmic reticulum (GO:0097038), which is located near the nucleus, and the peroxisomal membrane (GO:0005778), which surrounds peroxisomes. The nuclear replication fork (GO:0043596) is involved in DNA replication, while the small nuclear ribonucleoprotein complex (GO:0030532) plays a role in RNA processing. The RNA polymerase II transcription regulator complex (GO: 0090575) regulates gene expression. Chromatin (GO:0000785) and chromosomes are involved in DNA packaging and organisation (GO:0005694). The mitochondrion (GO:0005739) is responsible for generating energy in the form of ATP through cellular respiration, the nucleus (GO:0005634) houses the genetic material of the cell and controls cellular activities (GO:0005634), and the cytoplasm (GO:0005737). These cellular components play crucial roles in various cellular processes. The localisation of FATP1 within these components suggests that FATP1 may have important functions related to gene expression regulation, DNA packaging and organisation, energy generation, and overall cellular activities. Further investigation is needed to fully understand the specific role of FATP1 in each of these cellular compartments.

### 3.9. Pathway Enrichment Analysis of Target Proteins

The enriched KEGG pathway showed the involvement of FATP1 (listed in Table 2) in the thyroid hormone, PPAR, adipocytokine signalling, and other pathways. It was also involved in estrogen-dependent gene expression, nuclear receptor signalling, lipid metabolism, transcriptional regulation of white adipocytes differentiation, and transcriptional activation of mitochondrial biogenesis. These findings suggest that FATP1 may play a significant role in the development and progression of cancer through its involvement in various cellular processes such as hormone regulation, metabolism, and gene expression. Further research is needed to fully understand the exact mechanisms by which FATP1 influences cancer. Studying the specific pathways in which FATP1 is involved may provide insights into the underlying biology of cancer and help identify novel therapeutic targets.

## 4. Analysis of Gene–Disease Association

Data on gene–disease associations for a variety of illnesses, including rare diseases, are gathered by the DisGeNET resource. It ranks gene–disease associations according to a score, which is based on the quantity, quality, and type of sources and publications used to support the association. We gathered gene–disease associations from the DisGeNET resource (25 diseases) with gene–disease association scores of less than 0.2. Table 3 lists all genetic associations along with their scores.

## 5. Discussion

An evolutionarily conserved protein named fatty acid transport protein 1 (FATP1) localises to the plasma membrane to facilitate the transportation of fatty acids (FAs). This protein is essential for maintaining lipid homeostasis by promoting the uptake and use of fatty acids in many metabolic pathways. Its deficiency can result in poor energy metabolism and lipid accumulation in adipose tissue, potentially contributing to metabolic diseases like obesity, cancer, and insulin resistance [73]. The first in vivo study shows that FATPs regulate the amount of dietary lipids that are distributed throughout tissues, and insulin can regulate LCFA absorption by tissues through activating FATP1 [61]. Acyl CoA synthetase and FA transporter are both functions of FATP1 [11].

Although the crystallographic arrangement of the human FATP1 protein eluded discovery through the NCBI-BLASTp and PDB search tools, we resorted to modelling its structure via a sophisticated database tool known as ALPHAFOLD. ALPHAFOLD harnesses the power of deep learning algorithms to predict protein structures with remarkable precision. By scrutinising the amino acid sequence of FATP1, ALPHAFOLD successfully generated a three-dimensional representation of the protein’s structure, thereby imparting valuable insights into its functional attributes and potential interactions with other molecular entities [38]. In the context of our current investigation, we subjected the ALPHAFOLD-generated models to a rigorous assessment employing conventional evaluation methodologies, along with a 100 nanosecond molecular dynamics (MD) simulation. The outcomes of these analyses unequivocally substantiated the high quality and stability of the models. This substantiates the assertion that, beyond its distinguished performance in structural modelling competitions, ALPHAFOLD is adept at producing invaluable models for advanced scientific research.

After obtaining the FATP1 structure from ALPHAFOLD, the secondary structures were examined using the SOPMA PSIPRED servers [43,44], optimised, and refined using the galaxyrefine server. The 3D-modelled FATP1 enhanced the proportion of Ramachandran outliers, bad side chain rotamers, and low clash scores [45]. The HARMONY and ProSA web servers were used to further evaluate the final model, revealing its overall stability and quality. To identify potential local errors in areas where the reverse sequence had a higher substitution score, the HARMONY substitution graph provided scores between query sequences. The Dynamine server predicted backbone flexibility at the residue level in the form of a backbone. Regarding the NH S2 order parameter values, the atomic bond vector’s movement constraints in the molecular reference frame were reflected by these S2 values.

A coarse dynamics study was carried out using the CABS flex server to ascertain the residue level fluctuations about the protein’s most favourable conformation [77]. The 3D model of FATP1 was validated by PROCHECK [48], ERRAT [49], VERIFY 3D [50], and PROVE [51] tools from the structural analysis and verification server (SAVES). PROCHECK was used to assess the protein structure’s stereochemical quality. The VERIFY 3D tool determined if an atomic model (3D) and its amino acid sequence (1D) were consistent with each other. The ProSA web servers were utilised to host the anticipated models [45].

Out of three domains that were obtained during the secondary structure prediction (Figure 1A), the first domain was the signal peptide region, a short sequence of amino acids located at the N-terminus of a protein. It plays a crucial role in directing the protein to its correct cellular location. This region is recognised by the signal recognition particle (SRP), which helps in targeting the protein to the endoplasmic reticulum for further processing and secretion [78]. The AMP-dependent synthase/ligase domain (Domain II) appeared to function via an ATP-dependent covalent binding of AMP to its substrate. This domain is present in a number of prokaryotic and eukaryotic enzymes. The Ser/Thr/Gly-rich domain in this area is further distinguished by a conserved Pro-Lys-Gly triplet. This category of enzymes consists of acetyl-CoA synthetase, luciferase, long-chain fatty acid Co-A ligase, long-chain fatty acid transport proteins, and numerous other closely related synthetases. These enzymes play critical roles in various metabolic pathways and are essential for cellular function [79,80,81]. The AMP-binding enzyme (Domain III) was found in the C-terminal region (Figure 1A), indicating its crucial role in the enzymatic activity of the protein. This domain is responsible for binding AMP molecules and facilitating their conversion into a different form or catalysing a reaction involving AMP. The presence of this domain suggests that the protein may play a role in energy metabolism or signalling pathways related to AMP [82,83,84].

After validating the 3D model of the FATP1 structure, a protein stability analysis was conducted through molecular dynamic simulation. The MDS for the FATP1 refined structure was run in the current study for 100 ns, and the validated FATP1 structure showed that the RMSD trajectory reached equilibrium beyond 20 ns with a low mean square deviation range between 2.4 and 2.6 nm, which was maintained throughout the entire duration of the MDS when subjected to the MDS for 100 ns in an aqueous environment (Figure 3A). This indicates that the FATP1 structure was stable and did not undergo significant conformational changes during the simulation. The low RMSD values suggest that the protein remained in a compact and well-defined structure, which is essential for its proper functioning in biological processes. It was also stated that the average RMSF value, which denotes minimal atomic level positional variation, was between 0.05 and 1.0 nm (Figure 3B). This range indicates that the protein structure was relatively stable, with minimal fluctuations in atomic positions. The low RMSF values suggest a well-defined and compact protein conformation. The compactness of the protein structure was ensured by Rg’s low average value (2.6 nm) and steady trajectory (Figure 3C). The FATP1 potential energy curve (Figure 3D) also demonstrated a constant energy profile during the MDS, with a mean stability of 5.43 × 10^6^ kJ/mol. This indicates that the protein remained stable throughout the molecular dynamic simulation. 5.43 × 10^6^

After predicting the FATP1 structure by homology modelling, we then considered incorporating a network analysis because FATP1 played a role in the emergence of several diseases, including cancer. To establish the interconnection between FATP1 and the network of other genes involved in these diseases and to study the mechanisms and pathways through which FATP1 affects biological, cellular, and molecular processes, the protein was subjected to a string analysis after being validated through MDS-based simulation to gain insight into the protein–protein interaction. The analysis of strings included all genes, proteins, and neighbour interactions (Figure 4). The network of FATP1 with different proteins was revealed by an additional interactomic analysis. This interactomic analysis provided valuable insights into the potential functional associations and regulatory mechanisms of FATP1 in metabolic diseases. The interactions are depicted in (Figure 5), and an interactomics analysis of the hub gene showing 54 interactors and 55 interactions involved in the functional enrichment analysis of FATP1 is listed in Supplementary Tables S1 and S2.

According to its function, FATP1 participates in the FA metabolism, including esterification and oxidation. Insulin, PPAR activators, and transcription factors are additional energy-related elements that can regulate the expression of FATP1. These occurrences relate FATP1 to the cellular lipid synthesis [11]. Moreover, studies have shown that the dysregulation of FATP1 expression is associated with various metabolic disorders, such as obesity and insulin resistance and cancer. Therefore, targeting FATP1 may be a potential therapeutic strategy for these conditions. 

The GO enrichment analysis revealed GO terms for the biological process (BP), molecular function (MF), and cellular component (CC). These GO terms provided insights into the involvement of FATP1 in specific functions and processes, for example, the BP analysis showed enrichment (Table 1) in the regulation of lipid metabolism, cellular response to fatty acids, and positive regulation of the triglyceride biosynthetic process. Furthermore, the lipid localisation, hormone-mediated signalling pathway, lipid transport, regulation of fatty acid oxidation, response to progesterone, and regulation of transcription are some of the prominent biological processes that may demonstrate the involvement of FATP1 in the progression of various metabolic diseases, including breast cancer. Previous studies additionally demonstrate the involvement of FATP1 in cell proliferation [75]. These results indicate an intricate interplay between the FATP1 and a variety of lipid-related activities, hormone-mediated signalling pathways, and cellular responses to certain stimuli. The importance of FATP1 in metabolic diseases, including cancer, is made evident by its role in LCFA transport into cells, intracellular receptor signalling pathways, and the regulation of fatty acid oxidation. Since breast cancer is a hormone-sensitive cancer, the response to progesterone and other hormones highlights the importance of FATP1 in breast cancer development. Furthermore, research has demonstrated a link between high levels of FATP1 expression and tumor aggressiveness, underscoring the role of this protein in the development of hormone-sensitive breast cancer [10]. Overall, these activities imply that FATP1 may participate in the development of breast cancer by regulating lipid metabolism, responding to fatty acids, and promoting cell proliferation.

The MF analysis revealed enrichment in nuclear receptor transcription co-activator activity, transcription regulator activity, nuclear receptor binding, RNA polymerase II-specific DNA-binding transcription factor binding, arachidonate-CoA ligase activity, RNA polymerase II intronic transcription regulatory region sequence-specific DNA binding, and chromatin binding, which are some of the significant molecular activities listed in Supplementary Table S3. The described binding activities, including DNA and chromatin binding, imply that FATP1 is important in controlling gene expression. This is corroborated by the fact that it has RNA polymerase II-specific DNA-binding transcription factor binding activity, pointing to a possible function in regulating the transcription of particular genes. Apart from that, the activities of arachidonate-CoA ligase and long-chain fatty acid-CoA ligase imply that FATP1 is in charge of preparing these fatty acids for other metabolic pathways. Overall, FATP1′s multiple molecular functions and cellular components highlight its significance in numerous biological processes and give insight into its functional properties.

Lastly, the CC analysis indicated that FATP1 is primarily localised to the plasma membrane, highlighting its importance in cellular lipid uptake and transport. The PANTHER overrepresentation test identified 33 cellular components. These terms refer to various cellular compartments and structures, including chromatin and chromosomes, the perinuclear endoplasmic reticulum, the peroxisomal membrane, the nuclear replication fork, RNA processing, and the RNA polymerase II transcription regulator complex. The aforementioned components are essential for many biological functions. The localisation of FATP1 inside these components suggests that it may play significant roles in the regulation of gene expression, DNA packing and organisation, energy production, and other cellular processes. To completely comprehend the precise function of FATP1 in each of these cellular compartments, an additional investigation needs to be conducted.

The pathway enrichment analysis showed the involvement of FATP1 (Table 2) in the thyroid hormone signalling pathway, PPAR signalling pathway, fat digestion and absorption, adipocytokine signalling pathway, and prominent reactome pathways including (Table 2) estrogen-dependent gene expression (HSA-9029569), etc. Furthermore, the analysis revealed the significant involvement of FATP1 in several metabolic pathways, such as fatty acid metabolism and lipid transport. These findings suggest that FATP1 plays a crucial role in hormone signalling and metabolic regulation. Moreover, the enrichment analysis identified specific reactome pathways related to estrogen-dependent gene expression, indicating a potential link between FATP1 and estrogen-signalling pathways. Overall, these results highlight the multifaceted role of FATP1 in various cellular processes and provide insights into its functional significance.

Furthermore, the gene–disease association revealed the involvement of FATP1 in various diseases; some of the major diseases are listed in Table 3. FATP1 was found to be associated with obesity, insulin resistance, gestational diabetes mellitus, endometrial carcinoma, and breast cancer. These findings suggest that FATP1 plays a crucial role in lipid metabolism and may have significant implications in the development and progression of these diseases. Understanding the specific mechanisms by which FATP1 contributes to lipid uptake and transport could potentially lead to the development of targeted therapies for these conditions.

## 6. Conclusions

FATPs participate in several metabolic diseases, including cancer, through the FA signalling pathway. This pathway regulates the uptake, transport, and utilisation of FAs in cells. Abnormalities in FATPs have been linked to cancer, insulin resistance, and obesity. FATPs are thought to significantly contribute to the development of cancers like breast, prostate, bladder, liver, kidney, and lung cancer by promoting the proliferation, metastasis, and invasion of cancer cells. The inhibition of FATP expression or activity has been found to reduce tumour growth and enhance the efficacy of chemotherapy in preclinical models. To develop treatments that specifically target these FA transporters’ actions, a more in-depth understanding of their functions is required. FATP1 is notable among the six FATP isoforms because it has been linked to lipid accumulation in adipose tissue. The present study aimed to understand the dynamics of FATP1 contributing to numerous molecular associations. We predicted the structure of FATP1 in silico and thoroughly refined and validated it using coarse and molecular dynamics in the absence of the complete crystal structure. Their relative dynamics revealed the different properties of the characteristic FATP1.

The current study predicted that FATP1 would play a part in several gene ontology-based molecular functions, metabolic pathways, and biological processes. Further thought in the study was given to the possibility that intracellular signalling molecules might use FATP1-mediated pathways for cell survival and proliferation. The purpose of this study was to discover particular proteins in the interactome. We presented some additional information about the involvement of the FATP1/SLC27A1 gene in various diseases in the current study, which was based on the analysis of gene–disease data from the DisGeNET. For the advancement of translational and basic research on a variety of metabolic disorders like diabetes, obesity, gestational diabetes mellitus, insulin resistance, cardiovascular diseases, uterine fibroids, and primarily cancer, this information and examples may be helpful. However, to consider FATP1 as a potential therapeutic option for various metabolic diseases, its role in the development of the aforementioned metabolic diseases, including cancer, should be thoroughly investigated.

## Figures and Tables

**Table 1:** Biological process involved in functional enrichment analysis.

GO-TERM	Description	Count in Network	Strength	False Discovery Rate
GO:1904017	Cellular response to Thyroglobulin triodothyronine	2 of 2	3.25	0.0012
GO:1901700	Response to oxygen-containing compound	8 of 1567	0.96	0.00024
GO:1901576	Organic substance biosynthetic process	7 of 2734	0.66	0.0391
GO:0071396	Cellular response to lipid	5 of 528	1.23	0.0027
GO:0051173	Positive regulation of nitrogen compound metabolic process	8 of 3239	0.64	0.0166
GO:0048545	Response to steroid hormone	5 of 328	1.43	0.0005
GO:0071310	Cellular response to organic substance	8 of 2369	0.78	0.0026
GO:0045893	Positive regulation of transcription, DNA-templated	6 of 1587	0.83	0.0234
GO:0046320	Regulation of fatty acid oxidation	2 of 32	2.05	0.0341
GO:0045834	Positive regulation of lipid metabolic process	3 of 152	1.55	0.0205
GO:0045722	Positive regulation of gluconeogenesis	2 of 14	2.41	0.0108
GO:0045017	Glycerolipid biosynthetic process	3 of 229	1.37	0.0433
GO:0044539	Long-chain fatty acid import into cell	2 of 10	2.55	0.0067
GO:0044249	Cellular biosynthetic process	7 of 2611	0.68	0.0333
GO:0043401	Steroid hormone-mediated signalling pathway	4 of 118	1.78	0.00042
GO:0043393	Regulation of protein binding	3 of 212	1.4	0.0391
GO:0035336	Long-chain fatty-acyl-CoA metabolic process	2 of 25	2.15	0.0247
GO:0034654	Nucleobase-containing compound biosynthetic process	5 of 995	0.95	0.0287
GO:0034201	Response to oleic acid	2 of 6	2.77	0.0033
GO:0033993	Response to lipid	7 of 858	1.16	0.00011
GO:0033211	Adiponectin-activated signalling pathway	2 of 7	2.71	0.004
GO:0033036	Macromolecule localisation	8 of 2473	0.76	0.0033
GO:0032870	Cellular response to hormone stimulus	7 of 569	1.34	1.34 × 10^−5^
GO:0032570	Response to progesterone	2 of 45	1.9	0.048
GO:0031328	Positive regulation of cellular biosynthetic process	8 of 2005	0.85	0.0011
GO:0031325	Positive regulation of cellular metabolic process	9 of 3413	0.67	0.0027
GO:0030522	Intracellular receptor signalling pathway	4 of 166	1.63	0.0012
GO:0019432	Triglyceride biosynthetic process	2 of 20	2.25	0.0184
GO:0015908	Fatty acid transport	3 of 74	1.86	0.0038
GO:0019216	Regulation of lipid metabolic process	11 of 424	1.66	7.39 × 10^−15^
GO:0015721	Bile acid and bile salt transport	3 of 30	2.25	0.00057
GO:0015718	Monocarboxylic acid transport	6 of 142	1.88	4.85 × 10^−7^
GO:0014070	Response to organic cyclic compound	6 of 911	1.07	0.002
GO:0010906	Regulation of glucose metabolic process	3 of 118	1.66	0.0111
GO:0010876	Lipid localisation	7 of 326	1.58	5.16 × 10^−7^
GO:0010867	Positive regulation of triglyceride biosynthetic process	2 of 13	2.44	0.0099
GO:0010604	Positive regulation of macromolecule metabolic process	8 of 3600	0.6	0.0287
GO:0009755	Hormone-mediated signalling pathway	6 of 169	1.8	6.70 × 10^−7^
GO:0006869	Lipid transport	6 of 296	1.56	1.34 × 10^−5^
GO:0006629	Lipid metabolic process	5 of 1190	0.87	0.0457
GO:0006351	Transcription, DNA-templated	4 of 567	1.1	0.0391
GO:0006139	Nucleobase-containing compound metabolic process	7 of 2659	0.67	0.034
GO:1904017	Cellular response to Thyroglobulin triiodothyronine	2 of 2	3.25	0.0012
GO:1901700	Response to oxygen-containing compound	8 of 1567	0.96	0.00024
GO:1901576	Organic substance biosynthetic process	7 of 2734	0.66	0.0391
GO:0071396	Cellular response to lipid	5 of 528	1.23	0.0027
GO:0051173	Positive regulation of nitrogen compound metabolic process	8 of 3239	0.64	0.0166
GO:0048545	Response to steroid hormone	5 of 328	1.43	0.0005
GO:0045722	Positive regulation of gluconeogenesis	2 of 14	2.41	0.0108
GO:0071310	Cellular response to organic substance	8 of 2369	0.78	0.0026
GO:0045893	Positive regulation of transcription, DNA-templated	6 of 1587	0.83	0.0234
GO:0046320	Regulation of fatty acid oxidation	2 of 32	2.05	0.0341
GO:0045834	Positive regulation of lipid metabolic process	3 of 152	1.55	0.0205

**Table 2:** KEGG and reactome pathways involved in functional enrichment analysis of FATP1/SLC27A1.

KEGG Pathways Involved in Functional Enrichment Analysis				
GO-TERM	Description	Count in Network	Strength	False Discovery Rate
hsa04975	Fat digestion and absorption	2 of 41	1.94	0.0215
hsa04920	Adipocytokine signalling pathway	3 of 69	1.89	0.0013
hsa03320	PPAR signalling pathway	4 of 75	1.98	2.66 × 10^−5^
hsa04919	Thyroid hormone signalling pathway	3 of 119	1.65	0.0042
hsa04975	Fat digestion and absorption	2 of 41	1.94	0.0215
Reactome Pathways Involved in Functional Enrichment Analysis of FATP1/SLC27A1				
HSA-9623433	NR1H2 and NR1H3 regulate gene expression to control bile acid homeostasis	2 of 9	2.6	0.0012
HSA-9029569	NR1H3 and NR1H2 regulate gene expression linked to cholesterol transport and efflux	2 of 37	1.98	0.0137
HSA-9018519	Estrogen-dependent gene expression	3 of 119	1.65	0.0027
HSA-9006931	Signalling by nuclear receptors	4 of 265	1.43	0.00086
has-556833	Metabolism of lipids	11 of 733	1.43	4.39 × 10^−14^
HSA-4090294	SUMOylation of intracellular receptors	2 of 29	2.09	0.0093
HSA-3899300	SUMOylation of transcription co-factors	2 of 43	1.92	0.0174
HSA-383280	Nuclear receptor transcription pathway	2 of 53	1.83	0.024
HSA-3247509	Chromatin-modifying enzymes	4 of 237	1.48	0.0006
HSA-381340	Transcriptional regulation of white adipocyte differentiation	8 of 84	2.23	6.86 × 10^−15^
HSA-3214858	RMTs methylate histone arginines	2 of 49	1.86	0.0212
HSA-3108232	SUMO E3 ligases SUMOylate target proteins	4 of 166	1.63	0.00018
HSA-2426168	Activation of gene expression by SREBF(SREBP)	8 of 42	2.53	7.16 × 10^−17^
HSA-2151201	Transcriptional activation of mitochondrial biogenesis	8 of 54	2.42	3.75 × 10^−16^
HSA-211976	Endogenous sterols	3 of 27	2.3	6.07 × 10^−5^
HSA-1989781	PPARA activates gene expression	10 of 117	2.18	1.09 × 10^−18^
HSA-193807	Synthesis of bile acids and bile salts via 27- hydroxycholesterol	3 of 15	2.55	1.46 × 10^−5^
HSA-193368	Synthesis of bile acids and bile salts via 7alpha- hydroxycholesterol	3 of 24	2.35	4.62 × 10^−5^
HSA-159418	Recycling of bile acids and salts	3 of 16	2.52	1.63 × 10^−5^
HSA-1368108	BMAL1:CLOCK, NPAS2 activates circadian gene expression	8 of 27	2.72	3.93 × 10^−18^
HSA-1368082	RORA activates gene expression	8 of 18	2.9	1.05 × 10^−18^

**Table 3:** Gene–disease (SLC27A1/FATP1) association.

S.No.	Gene	Gene id	Disease	Disease id	Gene(SLC27A1)–Disease Association Score
1	SLC27A1	376497	Obesity	C0028754	0.22
2	SLC27A1	376497	Myocardial Infarction	C0027051	0.2
3	SLC27A1	376497	Hyperlipoproteinemias	C0020476	0.2
4	SLC27A1	376497	Hyperlipidemia	C0020473	0.2
5	SLC27A1	376497	Hyperinsulinism	C0020459	0.2
6	SLC27A1	376497	Impaired Glucose Tolerance	C0271650	0.2
7	SLC27A1	376497	Insulin Resistance	C0021655	0.2
8	SLC27A1	376497	Gestational Diabetes	C0085207	0.01
9	SLC27A1	376497	Endometrial Carcinoma	C0476089	0.01
10	SLC27A1	376497	Metabolic Syndrome X	C0524620	0.01
11	SLC27A1	376497	Breast Carcinoma	C0678222	0.01
12	SLC27A1	376497	Tumor Cell Invasion	C1269955	0.01
13	SLC27A1	376497	Photoreceptor Degeneration	C1998028	0.01
14	SLC27A1	376497	Experimental Organism Basal Cell Carcinoma	C3811653	0.01
15	SLC27A1	376497	Atherosclerotic Lesion	C4703473	0.01
16	SLC27A1	376497	Diffuse Large B-Cell Lymphoma	C0079744	0.01
17	SLC27A1	376497	Uterine Fibroids	C0042133	0.01
18	SLC27A1	376497	Cardiovascular Diseases	C0007222	0.01
19	SLC27A1	376497	Diabetes Mellitus, Non-Insulin-Dependent	C0011860	0.01
20	SLC27A1	376497	Fetal Growth Retardation	C0015934	0.01
21	SLC27A1	376497	Ichthyoses	C0020757	0.01
22	SLC27A1	376497	Congenital Ichthyosis	C0020758	0.01
23	SLC27A1	376497	Fibroid Tumour	C0023267	0.01
24	SLC27A1	376497	Melanoma	C0025202	0.01
25	SLC27A1	376497	Metabolic Diseases	C0025517	0.01
26	SLC27A1	376497	Malignant Neoplasm of Breast	C0006142	0.01
27	SLC27A1	376497	Carcinoma, Basal Cell	C4721806	0.01

## Data Availability

All the analysis that are used in the study is represented in the paper.

## Supplementary Materials

The following supporting information can be downloaded at: https://www.mdpi.com/article/10.3390/biom13111670/s1. Figure S1: The secondary structure prediction results for the FATP1 protein. The improved self-optimized prediction method (SOPMA) software was used to predict the secondary structure of the FATP1 protein. Which was largely made up of random coils (33.44%), followed by helix (34.98%), extended strand (22.91%), and turns (8.67%). Lines in different colors represent different secondary structures: Blue, α helix; green, β turn; red, extended strand; and purple, random coil. Fatty acid transport protien1; Figure S2: Substitution curve of FATP1, Fatty acid transport protien1; Table S1: Interactomics. analysis of hub gene showing 54 Interactors involved Functional enrichment analysis of FATP1; Table S2: Interactomic analysis of hub gene showing A Total of 55 Interactions involved Functional enrichment analysis of FATP1. Table S3: Molecular Functions involved in Functional enrichment analysis.

## Abbreviations

FATP1	Fatty Acid Transport Protein 1
LCFA	Long-Chain Fatty Acid
VLCFA	Very-Long-Chain Fatty Acid
SLC27A	Solute Carrier Family 27 A
COS1	Fibroblast-Like Cell Lines Obtained from Green Monkey Kidney Tissue
ACS	Acyl CoA Synthetase
BCC	Breast Cancer Cells
PDB	Protein Data Bank
AI	Artificial Intelligence
CASP	Critical Assessment of Structure Prediction
SOPMA	Self-Optimised Prediction Method with Alignment
PSI PRED	PSI-Blast Based Secondary Structure Prediction
RMSF	Root Mean Square Fluctuation
SAVES	Structural Analysis and Verification Server
MDS	Molecular Dynamic Simulations
