# Supplementary material for: AI-Based Homology Modelling of Fatty Acid Transport Protein 1 Using AlphaFold: Structural Elucidation and Molecular Dynamics Exploration
Source: Biomolecules. 2023 Nov 20;13(11):1670. doi: 10.3390/biom13111670 (PMC10669040; doi:10.3390/biom13111670)
Supplement: Supplementary file 1 [file biomolecules-13-01670-s001.zip › biomolecules-2653978-supplementary.pdf]

Supplementary material:

Table S1: Interactomics analysis of hub gene showing 54 Interactors involved Functional enrichment analysis of FATP1.

S. No.	Interactor	Organism	Aliases	Description
1.	RPE65	*Homo Sapiens*	rd12, LCA2, RP20, BCO3, sRPE65, mRPE65	Retinal pigment epithelium-specific protein 65kda
2.	ARMC6	*Homo Sapiens*	R30923_1	Armadillo repeat containing 6
3.	ARSF	*Homo Sapiens*	ASF	Arylsulfatase F
4.	ASIC4	*Homo Sapiens*	BNAC4, ACCN4	Acid-sensing (proton-gated) ion channel family member 4
5.	ATP2A1	*Homo Sapiens*	ATP2A, SERCA1	Atpase, Ca++ transporting, cardiac muscle, fast twitch 1
6.	C1ORF21[illegible]	*Homo Sapiens*	TEMP	Chromosome 1 open reading frame 210
7.	C3ORF52	*Homo Sapiens*	TTMP	Chromosome 3 open reading frame 52
8.	CA6	*Homo Sapiens*	CA-VI, GUSTIN, RP3-477M7.7	Carbonic anhydrase VI
9.	CD79A	*Homo Sapiens*	IGA, MB-1	CD79a molecule, Immunoglobulin-associated alpha
10.	CHRNA1	*Homo Sapiens*	FCCMS, SCCMS, CMS2A, CHRNA, ACHRA, ACHRD	Cholinergic receptor, Nicotinic, alpha 1 (muscle)
11.	CXCR3	*Homo Sapiens*	GPR9, MigR, Mig-R, CD183, CD182, CKR-L2, IP10-R, CMKAR3	Chemokine (C-X-C motif) receptor 3
12.	DGAT2	*Homo Sapiens*	ARAT, HMFN1045, GS1999FULL	Diacylglycerol O-acyltransferase 2
13.	DYM	*Homo Sapiens*	DMC, SMC	Dymeclin
14.	ERAP1	*Homo Sapiens*	ALAP, ARTS1, A-LAP, ERAAP, ERAAP1, ARTS-1,PILSAP APPILS	Endoplasmic reticulum aminopeptidase 1
15.	EVA1C	*Homo Sapiens*	B19, B18, SUE21, PRED34, FAM176C, C21orf64, C21orf63	Eva-1 homolog C (C. Elegans)
16.	FFAR1	*Homo Sapiens*	GPR40, FFA1R, GPCR40	Free fatty acid receptor 1
17.	FSD2	*Homo Sapiens*	SPRYD1	Fibronectin type III and SPRY domain containing 2
18.	GPR182	*Homo Sapiens*	AMR, AM-R, 7TMR, G10D, ADMR, gamrh, hrhAMR	G protein-coupled receptor 182
19.	HNRNPH[illegible]	*Homo Sapiens*	HNRPH, hnRNPH, HNRPH1	Heterogeneous nuclear ribonucleoprotein H1 (H)
20.	INS	*Homo Sapiens*	IDDM, ILPR, IRDN, IDDM2, IDDM1, MODY10	Insulin
21.	ITM2C	*Homo Sapiens*	E25, E25C, BRI3, ITM3, BRICD2C, hucep-14	Integral membrane protein 2C
22.	LDLRAD[illegible]	*Homo Sapiens*	-	Low-density lipoprotein receptor class A domain containing 1

23.	LGR4	*Homo Sapiens*	GPR48, BNMD17	Leucine-rich repeat-containing G protein-coupled receptor 4
24.	LPAR2	*Homo Sapiens*	EDG4, LPA2, LPA-2, EDG-4	Lysophosphatidic acid receptor 2
25.	LRRC55	*Homo Sapiens*	-	Leucine-rich repeat containing 55
26.	LRRC61	*Homo Sapiens*	HSPC295	Leucine-rich repeat containing 61
27.	LRRTM1	*Homo Sapiens*	UNQ675/PRO1309	Leucine-rich repeat transmembrane neuronal 1
28.	MDFI	*Homo Sapiens*	I-MF, I-mfa, RP4-696P19.1	Myod family inhibitor
29.	MPL	*Homo Sapiens*	MPLV, TPOR, CD110, C-MPL, THCYT2, RP1-92O14.1	MPL proto-oncogene, thrombopoietin receptor
30.	NUBP2	*Homo Sapiens*	CFD1, NUBP1, NBP 2	Nucleotide-binding protein 2
31.	OR7D2	*Homo Sapiens*	OR19-4, OR19-10, HTPCRH03	Olfactory receptor, family 7, subfamily D, member 2
32.	P3H4	*Homo Sapiens*	SC65, NO55, NOL55, LEPREL4	Prolyl 3-hydroxylase family member 4 (non-enzymatic)
33.	PBXIP1	*Homo Sapiens*	HPIP, RP11-307C12.2	Pre-B-cell leukaemia homeobox interacting protein 1
34.	PCDHGC4	*Homo Sapiens*	PCDH-GAMMA-C4	Protocadherin gamma subfamily C, 4
35.	PEX19	*Homo Sapiens*	PXF, PMP1, HK33, PMPI, PXMP1, PBD12A, D1S2223E,OK/SW-cl.22	Peroxisomal biogenesis factor 19
36.	PIGH	*Homo Sapiens*	GPI-H	Phosphatidylinositol glycan anchor biosynthesis, class H
37.	PLA2G10	*Homo Sapiens*	SPLA2, GXPLA2, GXSPLA2	Phospholipase A2, group X
38.	PRAMEF1	*Homo Sapiens*	-	PRAME family member 12
39.	PRSS8	*Homo Sapiens*	CAP1, PROSTASIN	Protease, serine, 8
40.	RNF4	*Homo Sapiens*	SLX5, SNURF, RES4-26	Ring finger protein 4
41.	SLC22A9	*Homo Sapiens*	OAT4, OAT7, ust3, HOAT4, UST3H	Solute carrier family 22 (organic anion transporter), member 9
42.	SLC39A4	*Homo Sapiens*	AEZ, ZIP4, AWMS2	Solute carrier family 39 (zinc transporter), member 4
43.	SYP	*Homo Sapiens*	MRX96, MRXSYP	Synaptophysin
44.	TAPBP	*Homo Sapiens*	TPN, TAPA, TPSN, NGS17, DADB-159G18.7	TAP binding protein (tapasin)
45.	TLR9	*Homo Sapiens*	CD289, UNQ5798/PRO19605	Toll-like receptor 9
46.	TOR1AIP	*Homo Sapiens*	NET9, LULL1, IFRG15, RP11-12M5.5	Torsin A interacting protein 2
47.	TPP1\	*Homo Sapiens*	LPIC, GIG1, CLN2, TPP-1, SCAR7	Tripeptidyl peptidase I
48.	TRIM67	*Homo Sapiens*	TNL	Tripartite motif containing 67
49.	TSPAN15	*Homo Sapiens*	NET7, NET-7, TM4SF15, 2700063A19Rik, UNQ677/PRO1311	Tetraspanin 15
50.	TTYH1	*Homo Sapiens*	XXbac-BCX535A19.1	Tweety family member 1
51.	TTYH3	*Homo Sapiens*	-	Tweety family member 3

52.	UBE4A	*Homo Sapiens*	E4, UFD2, UBOX2	Ubiquitination factor E4A
53.	ZDHHC12	*Homo Sapiens*	ZNF400, RP11-545E17.12-002	Zinc finger, DHHC-type containing 12
54.	ZNRF4	*Homo Sapiens*	spzn, RNF204, Ssrzf1, SPERIZIN	Zinc and ring finger 4

Table S2: Interactomic analysis of hub gene showing A Total of 55 Interactions involved Functional enrichment analysis of FATP1.

S. No.	Interactors	Organism	Experimental Evidence Code	Dataset	Throughput	HTP Score
1.	ARMC6	*Homo Sapiens*	Affinity Capture-MS	Huttlin EL (2021/pre-pub)	High	0.4723
2.	ARSF	*Homo Sapiens*	Affinity Capture-MS	Huttlin EL (2021/pre-pub)	High	0.7116
3.	ASIC4	*Homo Sapiens*	Affinity Capture-MS	Huttlin EL (2021/pre-pub)	High	0.5563
4.	ATP2A1	*Homo Sapiens*	Affinity Capture-MS	Huttlin EL (2021/pre-pub)	High	0.9202
5.	C1ORF210	*Homo Sapiens*	Affinity Capture-MS	Huttlin EL (2021)	High	0.9685
6.	C3ORF52	*Homo Sapiens*	Affinity Capture-MS	Huttlin EL (2021)	High	0.997
7.	CA6	*Homo Sapiens*	Affinity Capture-MS	Huttlin EL (2021)	High	0.9977
8.	CD79A	*Homo Sapiens*	Affinity Capture-MS	Huttlin EL (2021)	High	0.8251
9.	CHRNA1	*Homo Sapiens*	Affinity Capture-MS	Huttlin EL (2021)	High	0.9991
10.	CXCR3	*Homo Sapiens*	Affinity Capture-MS	Huttlin EL (2021/pre-pub)	High	0.8912
11.	DGAT2	*Homo Sapiens*	Affinity Capture-Western	Zhao YG (2017)	Low	-
12.	DYM	*Homo Sapiens*	Affinity Capture-MS	Huttlin EL (2021/pre-pub)	High	0.6949
13.	ERAP1	*Homo Sapiens*	Affinity Capture-MS	Huttlin EL (2021/pre-pub)	High	0.8383
14.	EVA1C	*Homo Sapiens*	Affinity Capture-MS	Huttlin EL (2021)	High	0.9083
15.	FFAR1	*Homo Sapiens*	Affinity Capture-MS	Huttlin EL (2021)	High	0.9413
16.	FSD2	*Homo Sapiens*	Affinity Capture-MS	Huttlin EL (2021/pre-pub)	High	0.8771
17.	GPR182	*Homo Sapiens*	Affinity Capture-MS	Huttlin EL (2021)	High	0.9056
18.	HNRNPH1	*Homo Sapiens*	Affinity Capture-RNA	Uren PJ (2016)	High	-
19.	INS	*Homo Sapiens*	Affinity Capture-MS	Tran DT (2020)	High	-
20.	ITM2C	*Homo Sapiens*	Affinity Capture-MS	Huttlin EL (2021)	High	0.7686
21.	LDLRAD1	*Homo Sapiens*	Affinity Capture-MS	Huttlin EL (2021)	High	0.9917
22.	LGR4	*Homo Sapiens*	Affinity Capture-MS	Huttlin EL (2021)	High	0.9328
23.	LPAR2	*Homo Sapiens*	Affinity Capture-MS	Huttlin EL (2021)	High	0.9143
24.	LRRC55	*Homo Sapiens*	Affinity Capture-MS	Huttlin EL (2021/pre-pub)	High	0.3696
25.	LRRC61	*Homo Sapiens*	Affinity Capture-MS	Huttlin EL (2021/pre-pub)	High	0.7056
26.	LRRTM1	*Homo Sapiens*	Affinity Capture-MS	Huttlin EL (2021/pre-pub)	High	0.7671
27.	MDFI	*Homo Sapiens*	Affinity Capture-MS	Huttlin EL (2021/pre-pub)	High	0.8855
28.	MPL	*Homo Sapiens*	Affinity Capture-MS	Huttlin EL (2021/pre-pub)	High	0.5039
29.	NUBP2	*Homo Sapiens*	Affinity Capture-MS	Huttlin EL (2021/pre-pub)	High	0.4835

30.	OR7D2	*Homo Sapiens*	Affinity Capture-MS	Huttlin EL (2021/pre-pub)	High	0.3996
31.	P3H4	*Homo Sapiens*	Affinity Capture-MS	Huttlin EL (2021/pre-pub)	High	0.6509
32.	PBXIP1	*Homo Sapiens*	Affinity Capture-MS	Huttlin EL (2021)	High	1
33.	PCDHGC4	*Homo Sapiens*	Affinity Capture-MS	Huttlin EL (2021/pre-pub)	High	0.985
34.	PEX19	*Homo Sapiens*	Affinity Capture-MS	Huttlin EL (2021)	High	0.9564
35.	PIGH	*Homo Sapiens*	Affinity Capture-MS	Huttlin EL (2021)	High	0.9921
36.	PLA2G10	*Homo Sapiens*	Affinity Capture-MS	Huttlin EL (2021/pre-pub)	High	0.8926
37.	PRAMEF12	*Homo Sapiens*	Affinity Capture-MS	Huttlin EL (2021/pre-pub)	High	0.3767
38.	PRSS8	*Homo Sapiens*	Affinity Capture-MS	Huttlin EL (2021/pre-pub)	High	0.6422
39.	RNF4	*Homo Sapiens*	Affinity Capture-MS	Kumar R (2017)	High	-
40.	RPE65	*Homo Sapiens*	Affinity Capture-Western	Guignard TJ (2010)	Low	-
41.	RPE65	*Homo Sapiens*	Reconstituted Complex	Guignard TJ (2010)	Low	-
42.	SLC22A9	*Homo Sapiens*	Affinity Capture-MS	Huttlin EL (2021)	High	0.7676
43.	SLC39A4	*Homo Sapiens*	Affinity Capture-MS	Huttlin EL (2021)	High	0.905
44.	SYP	*Homo Sapiens*	Affinity Capture-MS	Huttlin EL (2021)	High	0.9652
45.	TAPBP	*Homo Sapiens*	Affinity Capture-MS	Huttlin EL (2021/pre-pub)	High	0.5875
46.	TLR9	*Homo Sapiens*	Affinity Capture-MS	Huttlin EL (2021/pre-pub)	High	0.6826
47.	TOR1AIP2	*Homo Sapiens*	Affinity Capture-MS	Huttlin EL (2021/pre-pub)	High	0.9512
48.	TPP1	*Homo Sapiens*	Affinity Capture-MS	Huttlin EL (2021/pre-pub)	High	0.6289
49.	TRIM67	*Homo Sapiens*	Affinity Capture-MS	Demirdizen E (2022)	High	-
50.	TSPAN15	*Homo Sapiens*	Affinity Capture-MS	Huttlin EL (2021)	High	0.9936
51.	TTYH1	*Homo Sapiens*	Affinity Capture-MS	Huttlin EL (2021/pre-pub)	High	0.9973
52.	TTYH3	*Homo Sapiens*	Affinity Capture-MS	Huttlin EL (2021/pre-pub)	High	0.4954
53.	UBE4A	*Homo Sapiens*	Affinity Capture-MS	Huttlin EL (2021/pre-pub)	High	0.428
54.	ZDHHC12	*Homo Sapiens*	Affinity Capture-MS	Huttlin EL (2021)	High	0.8921
55.	ZNRF4	*Homo Sapiens*	Affinity Capture-MS	Huttlin EL (2021/pre-pub)	High	0.9257

Table S3. Molecular Functions involved in Functional enrichment analysis.

GO-TERM	Description	Count in network	Strength	False discovery rate
GO:0061629	RNA polymerase II-specific DNA-binding transcription factor binding	5 of 283	1.5	0.00046
GO:0140110	Transcription regulator activity	6 of 1657	0.81	0.0225
GO:0047676	arachidonate-CoA ligase activity	2 of 11	2.51	0.0066
GO:0030374	Nuclear receptor transcription coactivator activity	4 of 54	2.12	7.29E-05
GO:0017162	Aryl hydrocarbon receptor binding	2 of 9	2.6	0.0057
GO:0016922	Nuclear receptor binding	4 of 114	1.8	0.00046
GO:0004467	Long-chain fatty acid-CoA ligase activity	2 of 13	2.44	0.0081
GO:0003713	Transcription coactivator activity	5 of 316	1.45	0.00046
GO:0003682	Chromatin binding	4 of 570	1.1	0.0349
GO:0140110	Transcription regulator activity	6 of 1657	0.81	0.0225
GO:0001162	RNA polymerase II intronic transcription regulatory region sequence-specific DNA binding	2 of 13	2.44	0.0081
GO:0061629	RNA polymerase II-specific DNA-binding transcription factor binding	5 of 283	1.5	0.00046

Figures

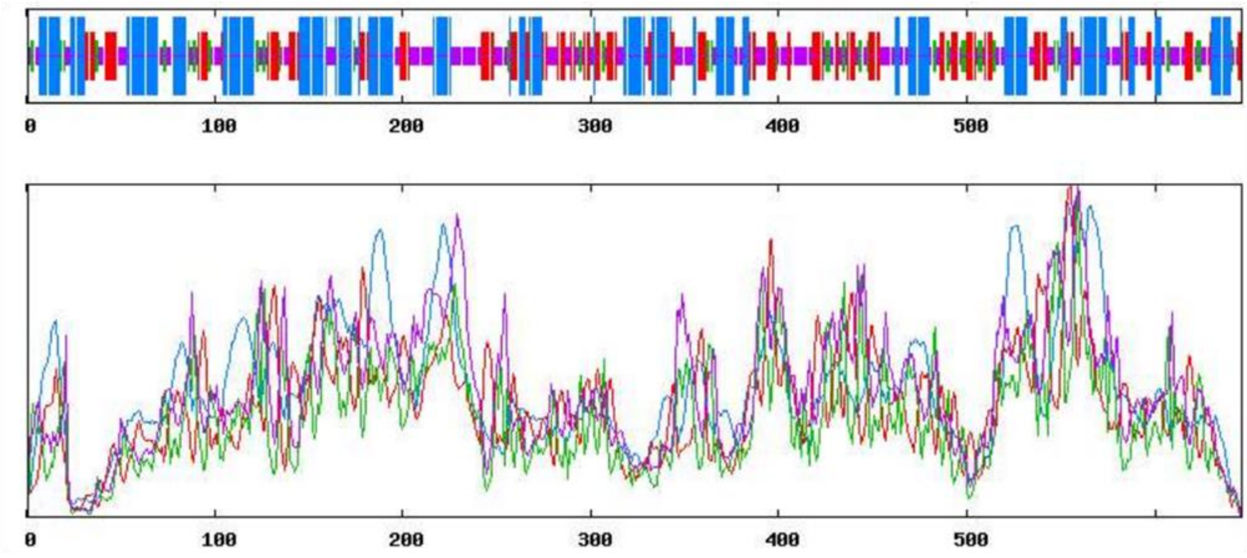

Figure S1: The secondary structure prediction results for the FATP1 protein. The improved self-optimized prediction method (SOPMA) software was used to predict the secondary structure of the FATP1 protein. Which was largely made up of random coils (33.44%), followed by helix (34.98%), extended strand (22.91%), and turns (8.67%). Lines in different colours represent different secondary structures: Blue, α helix; green, β turn; red, extended strand; and purple, random coil. Fatty acid transport protien1.

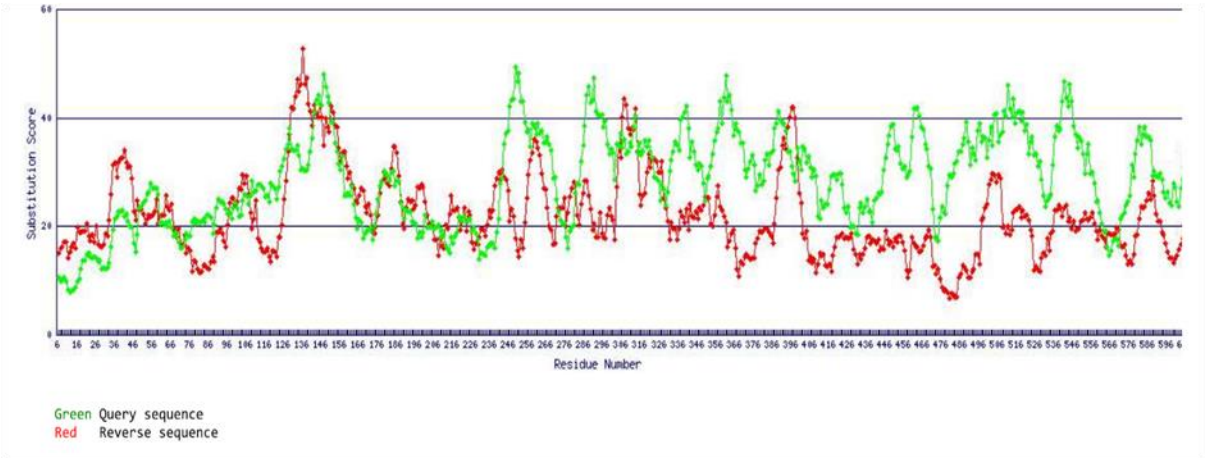

Figure S2: Substitution curve of FATP1, Fatty acid transport protien1
